# Antibiotic-Loaded Hydrogel for the Treatment of Lower-Limb Fracture-Related Infections: A Single Center’s Multidisciplinary Experience

**DOI:** 10.3390/gels10100628

**Published:** 2024-09-29

**Authors:** Daniele De Meo, Paolo Martini, Federico Lo Torto, Flavia Petrucci, Jessica Ordonez Reyna, Vittorio Candela, Giancarlo Iaiani, Alessandra Oliva, Diego Ribuffo, Stefano Gumina

**Affiliations:** 1 Department of Anatomical, Histological, Forensic Medicine and Orthopaedics Sciences, Sapienza University of Rome, 00100 Rome, Italy; paolo.martini@uniroma1.it (P.M.); jessicanicoleor@gmail.com (J.O.R.); vittorio.candela@uniroma1.it (V.C.); stefano.gumina@uniroma1.it (S.G.); 2 M.I.T.O. (Malattie Infettive in Traumatologia e Ortopedia—Infections in Traumatology and Orthopedics Surgery) Study Group, Policlinico Umberto I Hospital, Viale del Policlinico 155, 00161 Rome, Italy; federico.lotorto@uniroma1.it (F.L.T.); flavia.petrucci@uniroma1.it (F.P.); giancarlo.iaiani@uniroma1.it (G.I.); diego.ribuffo@uniroma1.it (D.R.); 3 Plastic Surgery Unit, Department of General Surgery, Plastic Surgeryand Orthopedics-Policlinico Umberto I Hospital-Sapienza, University of Rome, Viale del Policlinico 155, 00161 Rome, Italy; 4 Department of Public Health and Infectious Diseases, Sapienza University of Rome, 00100 Rome, Italy; alessandra.oliva@uniroma1.it; 5 Department of Internal Medicine, Endocrine-Metabolic Sciences and Infectious Diseases, Policlinico Umberto I University Hospital, 00161 Rome, Italy

**Keywords:** fracture-related infection, antibiotic-loaded hydrogel, lower-limb fracture, one-stage reconstruction, two-stage reconstruction, orthopedic trauma, local antibiotic delivery, bone healing, polymicrobial infection, multidrug-resistant pathogens

## Abstract

A fracture-related infection (FRI) is a severe complication of an orthopedic trauma, often leading to challenging treatments and poor outcomes. The surgical strategies are typically categorized into one-stage or two-stage procedures, with the use of systemic and local antibiotics being crucial for infection management. This study assessed the efficacy of an antibiotic-loaded hydrogel (ALH) applied over the internal fixation devices for treating FRIs, comparing the outcomes between the one-stage (OS) and two-stage (TS) reconstructions. This retrospective study included 17 patients with an FRI treated using the ALH at a single center. The patients were divided into OS and TS reconstruction groups. The data on demographics, surgical procedures, antibiotic regimens, and outcomes were collected. The primary and secondary outcomes included the infection cure rate, bone union, complications, and reoperation rates. Among the 17 patients (mean age 48.5 years, 16 males), infections were predominantly in the tibia, with 12 chronic and 5 acute cases. Seven patients had monomicrobial infections, and nine had multidrug-resistant pathogens. No significant differences were found between the OS and TS groups in terms of the infection cure rate, bone union, or complications. One patient in the OS group experienced an infection recurrence, and bone healing was achieved in all but one case. Additional complications included delayed wound closure in two cases and implant failure in one case, requiring a reoperation. The ALH demonstrated potential as an effective local antibiotic treatment for FRIs, particularly in the one-stage reconstructions, allowing for a safe application of internal fixation devices. However, further research with larger sample sizes and longer follow-ups is needed to validate these findings.

## 1. Introduction

A fracture-related infection (FRI) is a major complication of orthopedic trauma surgery, requiring multiple surgical procedures and antibiotic treatments. It is associated with difficult bone healing and poor functional outcomes. Furthermore, FRIs are linked to increased morbidity and amputation rates, significantly impacting the daily life and mental health of patients and their caregivers, as well as imposing an economic burden on the healthcare system [1,2,3].

Recent scientific advances have allowed for the standardization of diagnosis [4] and treatment, highlighting the importance of a multidisciplinary approach [5,6].

At present, we can divide the surgical treatment strategies into two groups: one-stage treatments and two-stage treatments. The first group includes debridement, antimicrobial therapy and implant retention (DAIR), and debridement and implant removal or implant exchange with a new osteosynthesis (one-stage reconstruction). The second group includes all surgical strategies that require two surgical interventions. The choice of the type of treatment depends on the general characteristics and comorbidities of the patient, their skin condition, the healing status and stability of the fracture, the size of the bone defect, the type of pathogen, and the time since diagnosis [5]. A fundamental issue associated with surgery is the use of systemic antibiotic therapy [7], although the concentrations that systemic antibiotics achieve in the bone are often variable and insufficient to effectively cure an infection. In this context, the use of local antibiotics can offer significant advantages, such as achieving higher local concentrations to rapidly reduce the microbial burden and protect the new implant without causing systemic side effects [8]. The use of local antibiotic delivery carriers is a useful tool for treating FRIs. The historically most widely used carrier is polymethyl methacrylate (PMMA) added with antibiotics, for its effective management of bone dead space [9,10]. Other types of local antibiotic carriers that have been used successfully are calcium–sulphate beads (CS) [11,12] and antibiotic-coated intramedullary (IM) nails [13].

Antibiotic-loaded hydrogels (ALHs) are a major focus of research within local delivery strategies, with the development of various technologies for both prophylaxis and treatment [14,15]. However, despite the potential benefits, there remains a limited amount of literature on the clinical application of ALHs. The available products authorized for clinical use include Expert Tibia Nail (ETN) PROtect^®^ (Synthes—Oberdorf, Switzerland) and Defensive Antibacterial Coating (DAC^®^Novagenit—Mezzolombardo, Italy). These tools could lead to the development of new surgical strategies for FRI treatment with internal fixation, with increased attention to the one-stage approach.

The primary aim of this study is to evaluate the efficacy and safety of using an ALH in the treatment of FRIs. This research seeks to establish this ALH as a valuable tool in the treatment of bone infections, given its ability to deliver high doses of local antibiotics, reducing re-infection rates without interfering with bone healing. As a secondary aim, this study also provides a comparative analysis of the outcomes when this ALH was employed within the context of one-stage reconstruction (OS) versus two-stage reconstruction (TS) techniques. Through this comparative evaluation, we aim to determine which surgical approach maximizes the therapeutic benefits of this ALH, offering critical insights that could shape future clinical practices for the management of septic orthopedic surgery. The goal was not only to address the safety of this ALH in an FRI setting, but to also assess the non-inferiority of an OS approach compared to a classic TS reconstruction.

## 2. Results and Discussion

### 2.1. Results

#### 2.1.1. Demographic Data

Seventeen patients were enrolled, with a mean age of 48.5 ± 15.6 years. Of these, 16 were male. The Mean Body Mass Index (BMI) was 24.81 ± 2.58, the Charlson Comorbidity Index (CCI) was 1.37 ± 1.54, and the American Society of Anesthesiologists (ASA) score was 2.17 ± 0.63. The infections were defined as acute in 5 cases, whereas 12 were chronic. A monomicrobial infection was present in seven patients, while the others were affected by a polymicrobial FRI; there was no culture-negative infection. An MDR pathogen was present in nine cases [Table 1]. The tibia was the bone most affected by an FRI (16 patients); the proximal tibia was affected in five cases, the diaphyseal tibia in two cases, the distal tibia in six cases, and malleolar fractures were present in three cases. One patient had a distal femur FRI [Table 2]. The patients were divided into two subgroups: a one-stage reconstruction group (OS) and a two-stage reconstruction (TS) group. In the OS group there were eight patients: all of them except one had internal fixation devices at the FRI diagnosis. Nine patients were treated with a TS reconstruction. No statistical differences were observed in terms of sex, age, BMI, CCI, ASA, timing, FRI classification [16], distribution of Gram-negative and -positive bacteria, or multidrug-resistant pathogens. A statistically significant difference was present among the distribution of polymicrobial infections within the two groups, with more polymicrobial FRIs in the TS group.

#### 2.1.2. Combined Treatment

The surgical and antimicrobial treatments are described in Table 3. The implants were changed in all cases but one, where the patient underwent a DAIR procedure because of an acute distal tibia FRI around a stable fixation and an anatomical reduction with good healing potential. In all the cases, the patients underwent internal fixation associated with hydrogel coatings. In five cases, a poly-d,l-lactic acid (PDLLA)-coated nail was also used. Vancomycin and gentamicin were used in all the cases. In one case, meropenem was also added to the hydrogel when treating a polymicrobial FRI with extended-spectrum-beta-lactamase (ESBL) *Klebsiella pneumoniae* with a one-stage revision. In twelve cases, bone grafting was performed, combining multiple techniques when needed: an iliac crest was used in six cases and in four cases the Reaming Irrigation Aspiration (RIA) system was applied. In three cases, a Free Vascularized Fibula Bone Graft (FVFBG) was used to fill the critical bone defect. A donor–cadaver bone graft was used only in two patients treated with the two-stage approach. Soft tissue plastic surgery reconstruction was needed in eleven patients, three in the OS reconstruction group and eight in the TS group. Systemic antibiotic therapy was carried out for a mean of 12.8 weeks, switching to oral delivery in eleven patients. Three patients underwent long-acting antibiotic treatment with dalbavancin.

#### 2.1.3. Outcomes

One patient had an FRI recurrence associated with a refracture, previously treated with a one-stage reconstruction with a plate, screws, and bone grafting of the medial malleolus after an ankle FRI [Table 4]. The patient was a diabetic who underwent a two-staged induced-membrane reconstruction technique that ended in an ankle arthrodesis with retrograde intramedullary nailing with iliac crest bone grafting to achieve bone healing. Four other complications occurred. In two cases, a delayed wound closure occurred, in both cases after an OS reconstruction was closed without the need of a plastic surgeon. In one case, a screw breakage occurred without the need for further treatment. In one case of a two-stage reconstruction of a distal tibia FRI treated with an induced membrane technique, the patient had an implant failure that needed a reoperation, in which a new plate was added in order to fortify the mechanical construction. Bone union occurred in all the cases but the infection recurrence one. No statistically significant differences were observed in terms of the infection cure rate (*p* = 0.4375), bone union rate (*p* = 0.4375), other complications (*p* = 0.6199), reoperation (*p* = 1), or the SF12 mental component score and physical component score (*p* = 0.1774 and *p* = 0.7787, respectively) between the two treatment groups.

### 2.2. Discussion

ALHs have been previously extensively described as a local prophylaxis in total hip replacements and revisions [17,18,19], highlighting their good results in reducing infection when also used in fracture fixation [20,21]. In a previously published narrative review, the potential effect of an ALH not only in a prevention setting but also in already infected cases was examined [22]. Up to date, this is the first report in which an ALH has been used as a local treatment for an FRI. In this setting, the rationale of its application was to protect the newly implanted implant from infection, either in a single-stage or multiple-stage reconstruction. Since the hydrogel completely resorbs in 48–72 h, its use relies on a sudden postoperative burst release. From the author’s perspective, the efficacy of the ALH was very well shown in the single-stage treatment, in which the surgical treatment has a pivot role if compared to TS reconstructions. The indications for a single-stage technique are actually disputed, but several review articles have cited it as a possibility when some conditions are present: acute cases with poor reduction or unstable fixation, and chronic cases with good soft tissue coverage and the absence of difficult-to-treat pathogens [5,23]. Anyway, the final decision is left to the treating multidisciplinary team, and especially to the surgeons and their confidence in debridement and external fixation skills. If the periprosthetic joint infection experience is applied to this setting, a one-stage revision leads to better functional results with the same infection eradication rate in selected cases [24]. In this study, all the patients were treated with internal fixation; the single-staged cases were the patients with an acute infection associated with poor reduction or unstable fixation, and chronic cases in which the patients did not have major bone defects or difficult-to-treat bacteria. The ALH was used to cover all the implants used in the one- or two-stage reconstructions in this study, but when an intramedullary nail was placed, a tibial nail with a poly(d,l-lactide) gentamicin coating was also used. This decision derived from two convictions of the authors. First, the need to use at least two types of local antibiotics in the FRI cases (ETN Protect has only gentamicin). Second, that the hydrogel could not be adequately applied onto a nail, because during the nail’s positioning through the nail entry point the hydrogel can be partially removed. Therefore, in the authors’ experiences, an ALH is useful to cover plates and screws, but its capacity to uniformly cover a nail cannot be assured. A gentamicin-coated nail has been already described as an internal fixation device in two-staged reconstruction procedures [25,26]. In that series, nine patients underwent a two-stage reconstruction with a coated nail and eight patients a single stage. None of them experienced infection recurrence.

In one-stage techniques, a conversion from an internal to an external fixation is usually carried out, to avoid the metal hardware being exposed to a risk of contamination. In a recent comparison of one- and two-staged treatments, ORIF was only used in 2.8% of the single-stage cases [27]. Single-stage treatment with internal fixation is described only when there is the possibility of a local antibiotic delivery, since today, implants are coated with PMMA to avoid their contamination [28,29]. There is now sufficient literature that shows that PMMA elutes its antibiotic in 7–10 days [30,31]. This technique also has some drawbacks, i.e., the dedicated time that is spent preparing the PMMA-coated nail or plate, and the greater volume of the coated plate, especially in a tibia FRI, which leads to more skin tension and a more difficult wound closure. These are some of the reasons why hydrogels have a potential role in this strategy.

If there are small bone voids that need to be addressed, antibiotic bone substitutes (i.e., calcium sulphate with hydroxyapatite) have already been described with success as a one-stage strategy in FRIs and osteomyelitis [32]. This type of ceramic cannot be used to cover the implant because it can be used only for bone defects. To protect the implant’s environment, antibiotic-loaded calcium sulphate beads are described only being used in acute settings and as a part of a DAIR technique in pelvic FRIs [33]. This is a promising approach to acute cases, but it is difficult to apply over subcutaneous implants where there is a higher risk of poor soft tissue coverage and subsequent wound leakage (i.e., anteromedial tibia shaft, ankle, olecranon).

The use of a local antibiotic delivery system is a useful adjunct, but it is not a substitute for a thorough debridement and soft tissue management that benefit from an orthoplastic approach associated with a proper systemic antibiotic treatment. Moreover, the key to the comprehensive and successful management of an FRI relies on an MDT approach.

This study has several limitations. The population is relatively small, but it must be considered in the light of the present literature, in which this is the first study that assesses the efficacy of this ALH in FRI treatment. This study’s design, its retrospective nature, and the absence of a control group impair the generalization of the results and makes it difficult to fully assess the independent effect of this ALH. Another limit to consider is the duration of the follow-up, conducted for a minimum of twelve months; even if it is sufficient to assess the bone healing process and the early onset of recurrence, it may not be sufficient to assess low-grade infection recurrence. Randomized clinical trials with lengthier follow-ups and greater sample sizes are, therefore, necessary in order to confirm the data from the present study. On the other hand, this study also presents with some strengths. First of all, this is the first study that investigates this ALH in FRIs, particularly comparing the standard two-stage approach with a one-stage surgery, which is now described for the first time. The data presented here are detailed and reproducible, laying out a solid basis for further research.

## 3. Conclusions

This study demonstrates the potential of this ALH as a useful part of an FRI multidisciplinary treatment, focusing on its role of protecting the implant during the reconstructive stage. This is the first clinical study in which DAC Gel is used to treat FRIs. Moreover, this study assesses the non-inferiority of an OS reconstruction with local delivery in selected cases compared to a TS surgery. This study’s limitations include the small sample size, the absence of a control group, and the short term of the follow-up. Future research should focus on larger randomized clinical trials with a longer follow-up to confirm the findings of this preliminary clinical experience.

## 4. Materials and Methods

### 4.1. Study Design

A single-center retrospective study was conducted by reviewing the electronic medical records of our hospital for patients admitted with a diagnosis of an FRI according to EBJIS/AO criteria [4]. We included all patients with an acute or chronic FRI diagnosis, treated with any kind of procedure in which the ALH was used in the reconstructive surgical stage, and with a minimum follow-up of 1 year. The exclusion criteria were patients treated with another type of local antibiotic carrier during the same surgical procedure, the presence of multiple localized infections, patients lost during the follow-up, and patients with an end-stage cancer diagnosis (prognosis <6 months). The preoperative anamnestic data, including the age, sex, comorbidities, CCI index, type of fracture according to the AO classification system, and the isolated pathogens and their antimicrobial resistance profiles, defined according to the classification of Magiorakos et al. [34], were collected from the electronic medical records. The intraoperative data on the surgical procedure, including the use of the ALH and the choice of local antibiotics, were collected from the electronic surgical register. We also recorded data on the length of stay, the duration and type of antibiotics, the follow-up, and the outcomes of infection and fracture healing.

This study was conducted in compliance with the ethical principles of the Declaration of Helsinki. Informed consent regarding the collection and analysis of surgery-related data was obtained from all the participants included in this study.

### 4.2. Treatment Strategy

All the patients were treated by the same multidisciplinary team, where the indications for a one- or two-stage procedure were discussed by the orthopedic and plastic surgeons and the infectious disease (ID) specialists together. All the patients underwent a preoperative microbiological biopsy, with the exception of septic patients whose surgery could not be postponed. The choice of a one- or two-stage reconstruction procedure was made in an MDT setting, taking into account the following: the fracture healing status, soft tissue conditions, patient’s comorbidities, timing of infection, and causative pathogen. All the patients underwent a preoperative CT scan to assess bone defects and vascularization. All the patients had a non-healed fracture. For acute FRIs, in the presence of a good reduction, stable construct, and possibility of a direct wound closure, the patients underwent a DAIR procedure. In acute cases, where there was a poor reduction without a bone defect or an unstable construct, but the possibility of direct wound closure, the patients underwent a one-stage reconstruction. Whenever, in acute or chronic cases, there was a poor and unstable reduction and/or insufficient soft tissue coverage, the patients underwent a two-stage procedure according to Masquelet [35]. During the first stage, the patients underwent debridement and hardware removal; a temporary reconstruction was performed by placing an antibiotic-loaded bone cement spacer into the bone defect and stabilized with an external fixation, covered with a local or free flap whenever a soft tissue defect was present. Systemic antibiotics were started during the first stage of the procedure, switching to an oral delivery whenever possible during the interstage [36]. Long-acting therapy was also considered, when applicable, to reduce the hospital stays of selected patients. During the second-stage surgical procedures, an internal fixation was performed; after spacer removal, the biological chamber was filled with a bone graft taken from various donor sites according to the preoperative evaluation.

The ALH used was DAC Gel (Novagenit, Mezzolombardo, Italy), a mixed composition of polylactic and hyaluronic acid re-hydrated with an antibiotic solution directly into the operative theater. The properties and possible uses of this hydrogel have been described previously [37]. The ALH comes in a 5 cc vial, in which a specific amount of gentamicin (100 mg) + vancomycin (125 mg) is used. In the cases in which meropenem was used, 2 vials (5 cc each) containing, respectively, gentamicin (100 mg) + vancomycin (125 mg) in one vial and 250 mg of meropenem in the second vial were prepared and then mixed together before coating.

In the cases in which a nail was used, a gentamicin-PDLLA-coated nail ETN Protect was used. The total amount of gentamicin embedded in one varies between 15 and 60 mg, depending on the size of the implant. The ALH was applied over the implant surface immediately before its insertion, and the choice of antibiotic was made by the surgeon and ID physician. The completion of at least twelve weeks of systemic antibiotics was achieved in all the cases; the extension of the length of the therapy was evaluated by the ID specialist case by case.

### 4.3. Statistical Analysis

The statistical analysis was performed with R version 3.4.4 (R Core Team (2018); R: A language and environment for statistical computing; R Foundation for Statistical Computing, Vienna, Austria). The categorical data were expressed as a percentage, while the continuous variables were reported as the mean and range. A two-sample *t*-test was used to compare the continuous variables when appropriate. Fisher’s exact test was used to compare the categorical variables. The level of significance was set at *p* < 0.05.

## Figures and Tables

**Table 1:** Population data. BMI: Body Mass Index; CCI: Charlson Comorbidity Index; ASA: American Society of Anesthesiologists; FRI: fracture-related infection; MDR: multidrug resistant.

	Total	One Stage	Two Stage	*p* Value
Age (mean ± SD)	48.5 ± 15.6	47.5 ± 12.9	55.1 ± 16	0.3021
Sex				0.4706
F	1	1	0	
M	16	7	9	
BMI (mean ± SD)	24.81 ± 2.58	25.67 ± 2.88	23.85 ± 2.12	0.1554
CCI (mean ± SD)	1.37 ± 1.54	1.3 ± 0.78	2.12 ± 1.72	0.2352
ASA (mean ± SD)	2.17 ± 0.63	2.3 ± 0.75	2.11 ± 0.60	0.5705
Timing				0.6199
Acute	5	3	2	
Chronic	12	5	7	
FRI Stage				0.7042
1	0	0	0	
2	3	2	1	
3	3	1	2	
4	11	5	6	
5	0	0	0	
Monomicrobial	7	6	1	0.0152
Polymicrobial	10	2	8	
Gram +	14	6	8	0.5765
Gram −	3	2	1	
MDR	9	2	7	0.0567

**Table 2:** Detailed demographical data. BMI: Body Mass Index; CCI: Charlson Comorbidity Index; ASA: American Society of Anesthesiologists; FRI: fracture-related infection; DAIR: debridement, antibiotic, implant retention.

Case	Age	Sex	BMI	CCI	ASA	Fracture (AO/OTA)	Gustilo Anderson	Timing (Acute/Chronic)	FRI Classification	Strategy
1	61	M	26.23	2	4	44B2.2	3b	chronic	4	one stage
2	63	M	26.45	4	2	44A2.1	N/A	chronic	4	two stage
3	84	M	23.15	4	3	43A3.3, 4F2B	N/A	chronic	2	two stage
4	50	M	30.6	0	2	41C3.3, 42C3	3b	chronic	4	one stage
5	34	M	22.5	0	2	41C3.3, 42C3	3B	acute	4	two stage
6	39	F	24.22	0	2	44A2.1	3b	acute	4	one stage
7	42	M	25	0	2	41A3.3	N/A	chronic	3	two stage
8	48	M	23.9	1	2	42B3, 4F2B	N/A	chronic	2	one stage
9	28	M	24.69	0	2	33B1.3	3b	acute	3	one stage
10	33	M	23.6	0	2	41A2.2	N/A	chronic	4	one stage
11	42	M	23.15	2	2	43C2.2	N/A	chronic	3	two stage
12	26	M	29.71	0	2	43C2.3, 4F2B	N/A	acute	2	one stage
13	43	M	24.22	2	2	43A1.2, 4F3A	2	chronic	4	DAIR
14	49	M	21	0	1	41C2.3	3A	chronic	4	two stage
15	71	M	26.2	2	2	43A3.3, 4F3A	2	chronic	4	two stage
16	50	M	21.2	1	2	43A3.1	2	chronic	4	two stage
17	61	M	26	4	3	42C2, 4F2B	3c	acute	4	two stage

**Table 3:** Detailed treatment data. MSSA: methicillin-susceptible *Staphylococcus aureus*; MSSE: methicillin-susceptible *Staphylococcus epidermidis*; CONS: Coagulase-negative *Staphylococci*; MRSE: methicillin-resistant *Staphylococcus epidermidis*; ESBL: extended-spectrum beta-lactamase; VRE: vancomycin-resistant *Enterococci*; DAIR: debridement, antibiotic, implant retention; RIA: Reaming Irrigation Aspiration; FVFBG: Free Vascularized Fibula Bone Grafting; DAC: Defensive Antibacterial Coating; ETN: Expert Tibia Nail; IV: intravenous; TMP/SMX: Trimethoprim/sulfamethoxazole.

Case	Pathogen	Strategy	Final Implant	Bone Grafting	Coating Strategy	Local Antibiotic	Soft Tissue Coverage	I.V. Antibiotic	Oral Antibiotic	LOS (Days)	ATB Treatment Duration (Weeks)
1	MSSA	one stage	Plate and screws	Proximal tibia	DAC	vancomycin, gentamicin	Sural flap	cefazolin	amoxicillin/clavulanic acid, rifampicin	35	16
2	*E. faecalis*, *C. striatum*	two stage	Intramedullary nail	Iliac crest, cadaver	DAC	vancomycin, gentamicin	None	linezolid	bacampicillin, linezolid	16	29
3	MSSE, CONS, *P. aeruginosa, C. albicans*	two stage	Intramedullary nail	RIA	DAC + ETN Protect	vancomycin, gentamicin	Sural flap	meropenem, amikacin, daptomycin, fluconazole		142	20
4	MRSE, *K. pneumoniae* ESBL	one stage	Intramedullary nail		DAC + ETN Protect	vancomycin, gentamicin, meropenem	Reversed flow hemisoleus flap	ertapenem, daptomycin		12	18
5	MRSE, *C. parapsilosis*	two stage	Total knee arthroplasty		DAC	vancomycin, gentamicin	Medial gastrocnemius flap	daptomycin, fluconazole		152	22
6	MSSA	one stage	Plate and screws		DAC	vancomycin, gentamicin	None	cefazolin	TMP/SMX, minocycline	14	4
7	MRSE, *Corynebacterium* spp.	two stage	Plate and screws	RIA, FVFBG	DAC	vancomycin, gentamicin	Medial gastrocnemius flap	daptomycin, rifampicin, dalbavancin	rifampicin	62	10
8	MSSA	one stage	Intramedullary nail		DAC + ETN Protect	vancomycin, gentamicin	None	amoxicillin/clavulanic acid, rifampicin	amoxicillin/clavulanic acid, rifampicin	2	8
9	*E. cloacae*, *S. maltophila*, *E. faecalis*	one stage	Screws + articulated external fixation	Iliac crest	DAC	vancomycin, gentamicin	Medial and lateral gastrocnemius flap	ceftazidime/avibactam, daptomycin		62	8
10	*Corynebacterium* spp.	one stage	Intramedullary nail	Iliac crest	DAC + ETN Protect	vancomycin, gentamicin	None	linezolid	linezolid	38	10
11	MRSE, CONS	two stage	Intramedullary nail	RIA, cadaver	DAC	vancomycin, gentamicin	Sural flap	daptomycin, dalbavancin		29	8
12	MSSA	one stage	Plate and screws	Iliac crest	DAC	vancomycin, gentamicin	None	TMP/SMX, minocycline	TMP/SMX, minocycline	20	17
13	MSSA	DAIR	Plate and screws		DAC	vancomycin, gentamicin	None	rifampicin, minocycline	rifampicin, minocycline	8	1
14	MSSA, *C. striatum*	two stage	Plate and screws	Iliac crest, fibula	DAC	vancomycin, gentamicin	Medial gastrocnemius flap	clindamycin, linezolid	clindamycin	43	10
15	MRSE	two stage	Plate and screws	Iliac crest, FVFBG	DAC	vancomycin, gentamicin	Sural flap	dalbavancin		8	6
16	CONS MR, *Candida albicans*	two stage	Plate and screws	FVFBG	DAC	vancomycin, gentamicin	Sural flap	daptomycin, fluconazole	fluconazole, minocycline	32	16
17	*Candida ciferrii*, VRE	two stage	Intramedullary nail	RIA, cadaver	DAC + ETN Protect	vancomycin, gentamicin	Sural flap	fluconazole, linezolid	fluconazole, linezolid	42	15

**Table 4:** Detailed outcomes data. DAIR: debridement, antibiotic, implant retention; SF: Short Form; MCS: mental component score; PCS: physical component score.

Case	Strategy	Bone Union	Infection Recurrence	Other Complications	Reoperation	SF-12 * MCS	SF-12 * PCS
1	one stage	No	Yes	Implant failure	Ankle arthrodesis	65.7	46.3
2	two stage	Yes	No	No	No	65.7	46.3
3	two stage	Yes	No	No	No	54.1	19.9
4	one stage	Yes	No	No	No	60.9	46.3
5	two stage	Yes	No	No	No	41.7	49
6	one stage	Yes	No	No	No	58.7	51.7
7	two stage	Yes	No	Screw breakage	No	57.7	51.8
8	one stage	Yes	No	No	No	52.7	47.5
9	one stage	Yes	No	No	No	41.7	45.1
10	one stage	Yes	No	No	No	63.4	49
11	two stage	Yes	No	No	No	58.1	45.1
12	one stage	Yes	No	Delayed wound closure	No	63.4	49
13	DAIR	Yes	No	Delayed wound closure	No	64.2	40.3
14	two stage	Yes	No	No	No	58.1	51.7
15	two stage	Yes	No	Plate breakage	Re-osteosynthesis	62	42.9
16	two stage	Yes	No	No	No	55.9	56
17	two stage	Yes	No	No	No	58	49.4

* 12 months follow-up.

## Data Availability

The original contributions presented in this study are included in the article.
